# Examining the When and How of Physical Activity Patterns in Older Adults: The Women’s Health Study

**DOI:** 10.1093/geroni/igaa057.2864

**Published:** 2020-12-16

**Authors:** Eric Shiroma

**Affiliations:** National Institute on Aging, Bethesda, Maryland, United States

## Abstract

Using data from the Women’s Health Study (N = 17,708, mean age = 72 years), we investigated the relative importance of physical activity volume, intensity, frequency of sessions, and session duration. We calculated the mortality hazard ratio for varying patterns and combinations of physical activity intensities and sedentary behavior. In separate analyses, we compared participants who engaged in activity regularly throughout the week to those who focused on one or two days a week, and the relative importance of session frequency and duration. These characteristics of activity patterns and their impact on health may help design interventions and public policy.

